# Sutured Cartilage Graft for Middle Ear Prosthesis Stabilization: A Case Report

**DOI:** 10.7759/cureus.87660

**Published:** 2025-07-10

**Authors:** Francisco Javier Mancilla Mejía, Jesus Alejandro Saenz Medina, Alfredo Vega Alarcon, Luis Miguel Méndez Saucedo

**Affiliations:** 1 Otorhinolaryngology and Head and Neck Surgery, Universidad Naval, Mexico City, MEX; 2 Otorhinolaryngology and Head and Neck Surgery, Universidad Autónoma de Nuevo León, Monterrey, MEX; 3 Neurotology, Instituto Nacional de Neurologia y Neurocirugia, Mexico City, MEX

**Keywords:** cartilage graft, chronic otitis media, ossiculoplasty, porp, prosthesis extrusion, torp

## Abstract

Prosthesis extrusion following ossicular chain reconstruction remains a common complication with a variable reported incidence. Interposition cartilage grafts are commonly used to minimize this risk, yet displacement still occurs. We describe a novel surgical technique in which a tragal cartilage graft is sutured directly to the prosthesis before placement, aiming to improve stability and reduce extrusion risk. A 37-year-old female with chronic otitis media and tympanic membrane retraction underwent ossiculoplasty. A partial ossicular replacement prosthesis was used. Prior to implantation, an autologous tragal cartilage graft was anchored to the prosthesis using two 9-0 nylon sutures in mirror configuration. The assembly was then positioned in the middle ear through an endoscopic transcanal approach. Follow-up at one week and one month demonstrated no evidence of extrusion, displacement, or complications. This technique offers a simple, low-cost, and effective modification to conventional ossiculoplasty. Suturing the cartilage graft to the prosthesis may enhance mechanical stability. Further studies are needed to evaluate long-term outcomes.

## Introduction

Extrusion of middle ear prostheses is a well-documented complication in ossicular reconstruction, with reported rates between 0% and 35% [1]. Canzi et al. reported extrusion or displacement in 5.2% of cases [1]. To address this, various surgical strategies have emerged, including the use of overlay cartilage grafts, which may reduce contact-induced inflammation between the prosthesis and tympanic membrane.

Mohseni-Dargah et al. reported failure rates up to 50% following ossiculoplasty, highlighting the need for alternative techniques to increase prosthesis stability [2]. Prosthesis failure can be attributed to transoperative factors, postoperative displacement, material incompatibility, or underlying middle ear pathologies such as Eustachian tube dysfunction.

Carey et al., in a review of passive middle ear implants, found device-related adverse events in 50% of partial ossicular replacement prosthesis (PORP) cases and 61.8% of TORP cases, with extrusion accounting for 78% of complications in the TORP group [3].

Despite numerous innovations, no prior report describes the use of sutures to secure a cartilage graft directly to the prosthesis before implantation. This report introduces a simple modification to the conventional technique to address this gap.

## Case presentation

**Figure 1:**
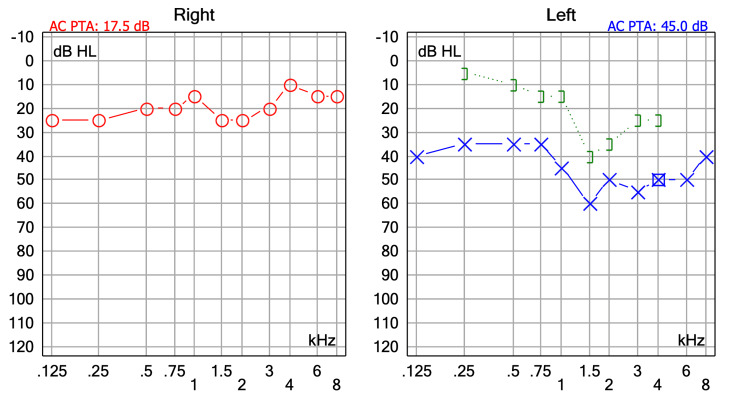
Preoperative audiometry showing moderate mixed hearing loss in the left ear with a pure-tone average of 45 dB

A 37-year-old female with no relevant medical history presented with a 10-year history of intermittent left aural fullness accompanied by progressive ipsilateral hearing loss. She denied otorrhea, vertigo, or previous ear surgeries. Otomicroscopic examination revealed a Grade III tympanic membrane retraction, with an attic pocket on the left side. The tympanic membrane was intact, without perforation or active infection. Audiometry demonstrated a moderate conductive hearing loss consistent with the patient’s clinical symptoms (Figure 1).

Temporal bone CT demonstrated soft tissue density at the level of the oval window, enveloping the long process of the incus and suggesting adhesion of the retracted tympanic membrane in that area. No ossicular chain dislocation or cholesteatoma was identified.

The patient was scheduled for left endoscopic ossiculoplasty. Under local anesthesia with sedation, a transcanal endoscopic approach was performed. Intraoperative endoscopic assessment revealed fibrous adhesions around the stapes and incus, with lysis of the long process and lenticular apophysis, consistent with focal ossicular erosion that was not evident on imaging. The middle ear mucosa appeared healthy. Given the extent of incus erosion and preservation of the stapes superstructure, a PORP (MED-EL, Innsbruck, Austria) was selected for reconstruction.

**Figure 2:**
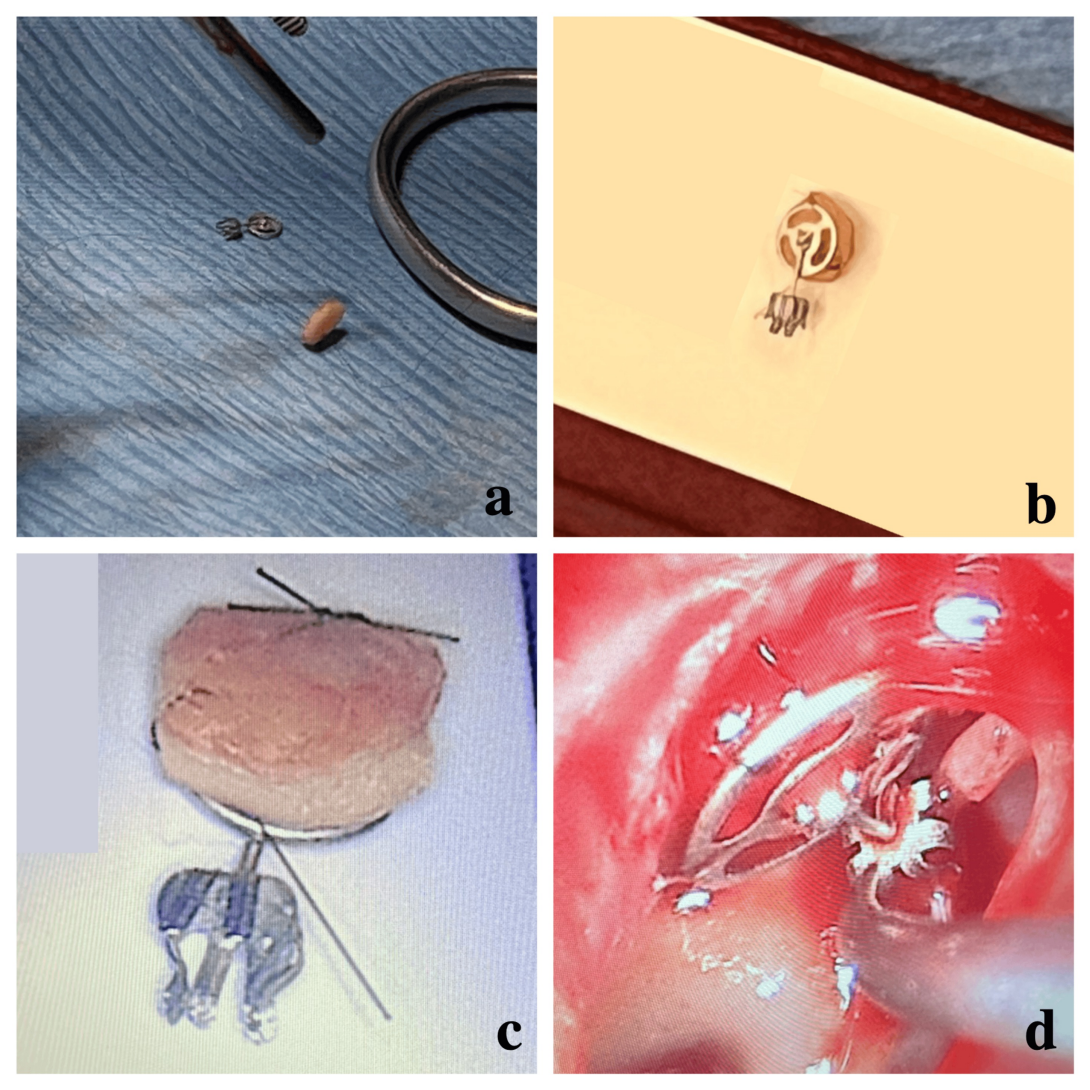
Intraoperative preparation and placement of the cartilage-prosthesis unit (a) Harvested and trimmed tragal cartilage. (b) 9-0 nylon monofilament sutures passed through both cartilage and prosthesis. (c) Completed assembly with cartilage fixed to the prosthesis. (D) Final endoscopic positioning in the middle ear.

An autologous tragal cartilage graft was harvested from the ipsilateral side, with perichondrium removed (Figure 2a). The cartilage was trimmed to match the prosthesis head (Figure 2b). To improve prosthesis stability, two 9-0 nylon monofilament sutures were passed through the prosthesis and the cartilage in a mirror configuration, anchoring the two elements firmly together (Figure 2c). The assembly was placed in the middle ear with the cartilage facing laterally toward the tympanic membrane (Figure 2d). The tympanomeatal flap was repositioned, and no additional cartilage grafts were needed for tympanic membrane reconstruction, as the membrane remained structurally viable. Gelfoam^®^ was applied in the external auditory canal for support.

**Figure 3:**
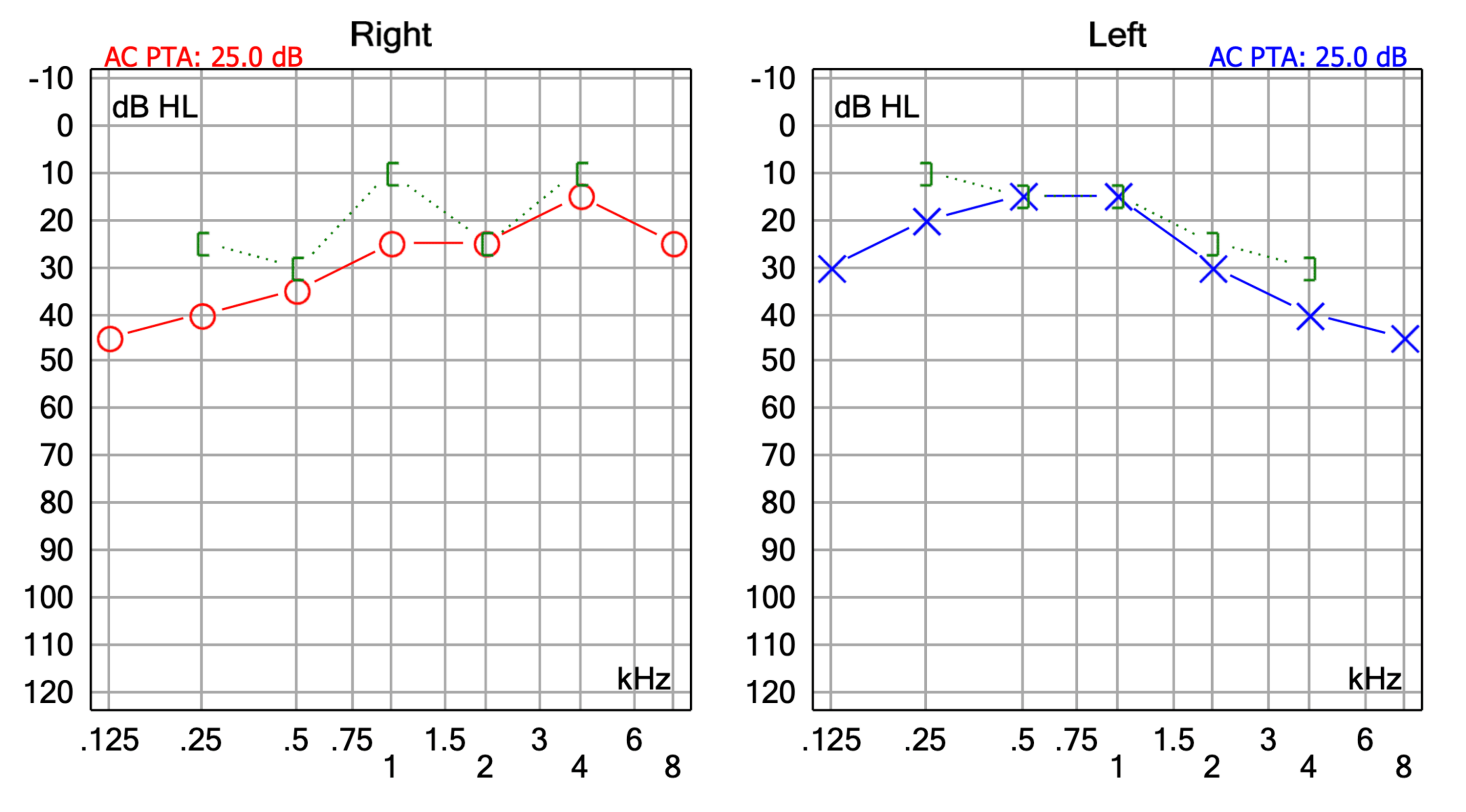
Postoperative audiometry at one month showing hearing improvement in the left ear with a pure-tone average of 25 dB

At the one-week follow-up, the patient reported no complications. Examination revealed intact tympanic membrane positioning, minor crusting in the canal, and no extrusion. At one month, endoscopic otoscopy confirmed a well-positioned prosthesis and stable cartilage graft, with no signs of medialization, lateralization, or extrusion. The tympanic membrane appeared healed and intact. The patient reported subjective improvement in hearing, which was confirmed by postoperative audiometry, showing a 20 dB improvement in pure-tone average, consistent with the clinical outcome and indicative of successful ossicular reconstruction (Figure 3).

## Discussion

Cartilage interposition between the prosthesis and tympanic membrane has been proposed to mitigate inflammation and improve long-term outcomes. However, Mosconi et al. note conflicting evidence in the literature and emphasize surgical technique as a potential confounder [4]. Lahlou et al., in a retrospective study of 256 patients, used cartilage overlay in 187 cases and reported 3% extrusion and 6% dislocation at one year [5]. Filosa et al. reported superior stability with a double cartilage block technique (Malafronte method) compared to incus interposition [6].

Innovations include Mosconi’s biohybrid prosthesis with a bone-derived extracellular matrix and Erdim’s three-part cartilage-PORP composite design [4,7]. Fink et al. reported similar complication rates regardless of surgical approach (endoscopic vs. microscopic) [8]. No ossiculoplasty material has been definitively proven superior, but autologous cartilage remains favorable due to its biocompatibility and stability [9]. Alwabili et al. identified isotretinoin use as a risk factor for graft thinning and extrusion, recommending postponement of surgery for six to 12 months after administration [10]. Mocanu et al. emphasized prosthesis design flaws as contributors to failure, supporting the rationale for improved mechanical fixation, such as the use of sutures to anchor grafts [11].

We hypothesize that shearing forces from middle ear pressure fluctuations or Eustachian tube dysfunction may be better tolerated with this technique. Based on this rationale, fixing cartilage to a titanium middle ear prosthesis using microsutures may offer several advantages, particularly in reconstructive otologic surgery, as it appears to enhance mechanical stability, promote tissue integration, and potentially reduce postoperative complications, including prosthesis extrusion, without increasing surgical complexity.

Given the inherently smooth surface of titanium prostheses, interposed grafts may be prone to slippage. This technique, which involves suturing the cartilage to the prosthesis, seeks to mitigate such displacement and ensure better integration and alignment. If revision surgery is required, the suture points may facilitate precise access and controlled manipulation of the graft-prosthesis complex. The use of non-absorbable monofilament sutures such as nylon 9-0 offers lasting fixation, while absorbable sutures may be preferred when tissue remodeling is anticipated.

Patients should be monitored closely to detect early signs of prosthesis migration or infection, such as persistent conductive hearing loss, recurrent otorrhea, tympanic membrane retraction, or visible graft displacement on otoscopy, which underscores the value of meticulous intraoperative technique. The surgeon’s familiarity with both middle ear anatomy and microsuturing technique directly influences the long-term success of prosthesis integration.

To our knowledge, no prior publication has described direct suturing of a cartilage graft to a prosthesis as a strategy to prevent extrusion. However, these findings should be interpreted with caution, as they are based on a single case report. Further research, including prospective studies with larger cohorts and longer follow-up, is needed to validate the long-term safety and efficacy of this technique.

## Conclusions

Fixing cartilage to a titanium prosthesis using microsutures may enhance prosthesis stability and promote better graft integration, potentially reducing the risk of extrusion or displacement. The described technique presents a promising method to reduce the displacement of cartilage grafts and prostheses in ossiculoplasty. It is simple, reproducible, and low-cost and does not appear to increase surgical risk. While the short-term outcome in this case was favorable, further case series or prospective studies are needed to validate its long-term effectiveness.
